# A consistent Great Lakes ice cover digital data set for winters 1973–2019

**DOI:** 10.1038/s41597-020-00603-1

**Published:** 2020-08-06

**Authors:** Ting-Yi Yang, James Kessler, Lacey Mason, Philip Y. Chu, Jia Wang

**Affiliations:** 1 Cooperative Institute for Great Lakes Research (CIGLR), University of Michigan, Ann Arbor, Michigan USA; 2 Geodetic Science, School of Earth Sciences, The Ohio State University, Columbus, Ohio USA; 3 Great Lakes Environmental Research Laboratory, National Oceanic and Atmospheric Administration, Ann Arbor, Michigan USA

**Keywords:** Cryospheric science, Limnology

## Abstract

Ice formation and loss in the Laurentian Great Lakes has a strong impact on regional climate, weather, economy and ecology in North America. To record the ice changes during the winter season, Great Lakes ice cover data has been collected and maintained since 1973 by Canadian Ice Service, U.S. National Ice Center, and National Oceanic and Atmospheric Administration’s Great Lakes Environmental Research Laboratory. Throughout this long history, technology has improved and the needs of users have evolved, so Great Lakes ice cover datasets have been upgraded several times in both spatial and temporal resolutions. In order to make those long-term data consistent and accessible, we reprocessed the Great Lakes ice cover database to generate daily gridded data (1.8 km resolution) using a re-project method with Nearest Neighbor Search for spatial interpolation, and linear interpolation with categorization for temporal interpolation. This report elucidates data history, generation procedures, and file structure in order to improve access and usability of Great Lakes ice cover data.

## Abstract (summary)

**Table:** 

Measurement(s)	ice-covered lake
Technology Type(s)	computational modeling technique • digital curation
Factor Type(s)	year of data collection
Sample Characteristic - Environment	climate system • lake • ice
Sample Characteristic - Location	Great Lakes

Machine-accessible metadata file describing the reported data: 10.6084/m9.figshare.12505388

## Background & Summary

The Great Lakes ice cover^1^ (GLIC) data are analysed and generated jointly by Canadian Ice Service (CIS) and U.S. National Ice Center (USNIC) using coordinated ice charting practices since winter 1973 and 1989, respectively (winter 1973 refers to the winter period in 1972–1973, the definition remains the same through the article). Their analyses are mainly based on integration of Synthetic Aperture Radar (SAR), visible and infrared imagery, and meteorological data, to represent the ice coverage at 1800 UTC^2^. The comprehensive description of ice cover observations is depicted in the USNIC Daily Ice Concentration Metadata^3^ and the CIS product handbook^1^. Great Lakes Environmental Research Laboratory’s (GLERL) role for GLIC is gathering and re-processing the data produced by CIS and USNIC in order to provide coherent and easily-available datasets for users, as well as to promote the GLIC database for use in diverse applications and scientific research.

**Fig. 1:**
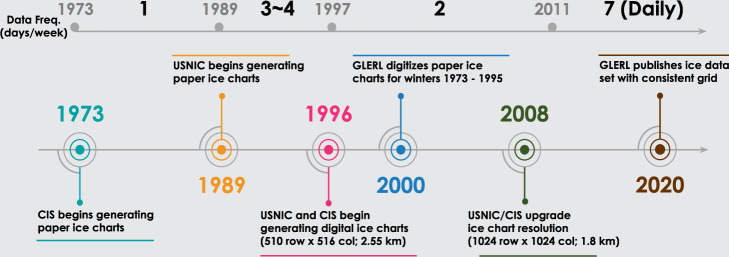
Timeline of ice chart evolution and frequency. During the winter 1989–1995, CIS and USNIC were reporting data independently, causing inconsistent ice estimations in the open water. CIS data consistently overestimate ice compared to USNIC, which periodically makes the data appear like ice is growing/melting every week. We decided to only include the USNIC data during this time, which means only three grids per week were available for these years. The inconsistency between CIS and USNIC was fixed in winter 1996, so the CIS data was included and the data frequency became 4 days per week during winter 1996–1997.

With the advancement of instrumentation equipment and varying user requirements, there are three major changes, outlined below, in GLIC data over the 46 year period of record. The overall timeline and data frequency are shown in Fig. 1.

From 1973 to 1995, data was recorded on paper ice charts. CIS generated one ice chart per week starting in winter 1973, and USNIC began producing complementary ice charts in the winter of 1989 at the rate of 3 to 4 days per week. All of these ice charts were recorded on paper until the winter of 1996. During the years 1995 through 1998, a team of GLERL researchers digitized paper ice charts for the winters of 1973 to 1995^4^ by 1) digitizing ice charts on a standardized Great Lakes shoreline base map; 2) transforming the ice information from World Meteorological Organization (WMO) codes to GLERL’s numerical form; and 4) generating 510 × 516 grid ASCII files.

Starting in winter 1996, USNIC and CIS began generating ice charts in digital form on a 510 × 516 grid (2.55 km resolution)^5^. This improved searchability and data access. In winter 1997, the data frequency changed to 2 days per week (one from CIS and one from USNIC), and continued with this frequency until winter 2010. In winter 1999, USNIC and CIS began to exchange their ice analysis, and USNIC has since collected and uploaded all of the ice charts to their public website^3^ for establishing a comprehensive ice atlas. Coinciding with this consolidation of ice data hosting, GLERL began using USNIC exclusively as an ice data source^6^.

In winter 2008, due to advancement of remote sensing and computer technologies, USNIC and CIS decided to upgrade the data spatial resolution from 2.55 km to 1.8 km^7^. For subsequent winters, the digital 1024 × 1024 ice cover grid has been provided. In addition, the map projection and Great Lakes water mask were also updated for the new ice atlas. Per GLERL’s request, USNIC/CIS started to produce daily ice charts (4 days from CIS, and 3 days from USNIC) in winter 2011. This resulted in reducing the uncertainty of ice data for the needs of scientific and commercial use^8^.

Therefore, in the preexisting GLIC data set, the spatial resolutions, projections, and sampling frequency of ice cover data vary throughout time. To standardize the spatial resolution, Wang *et al*.^7^ developed software to transpose the 510 × 516 grid (Grid-510) to 1024 × 1024 grid (Grid-1024). Their method ensures less than 2% spatial differences before and after transformation. However, Wang *et al*. only processed ice data for the winter 2007, and their resampling results have significant shifting error compared with original Grid-510. In addition, Wang *et al*. did not resolve inconsistent temporal resolution problems. Here, we are presenting a new, standardized GLIC data set created by: 1) standardization of spatial resolution throughout time (via transposing the data for winter 1973 to 2007 to the spatial grid of 2008-present), and 2) standardization of temporal resolution (via linear temporal interpolation to generate pseudo-daily ice cover data from winter 1973 to 2010). In the methods section, we describe the spatial and temporal interpolation methods used for standardization. In the technical validation section, we compare our results with other spatial interpolation methods (Kriging, bi-linear, and Wang *et al*.’s results).

## Methods

**Fig. 2:**
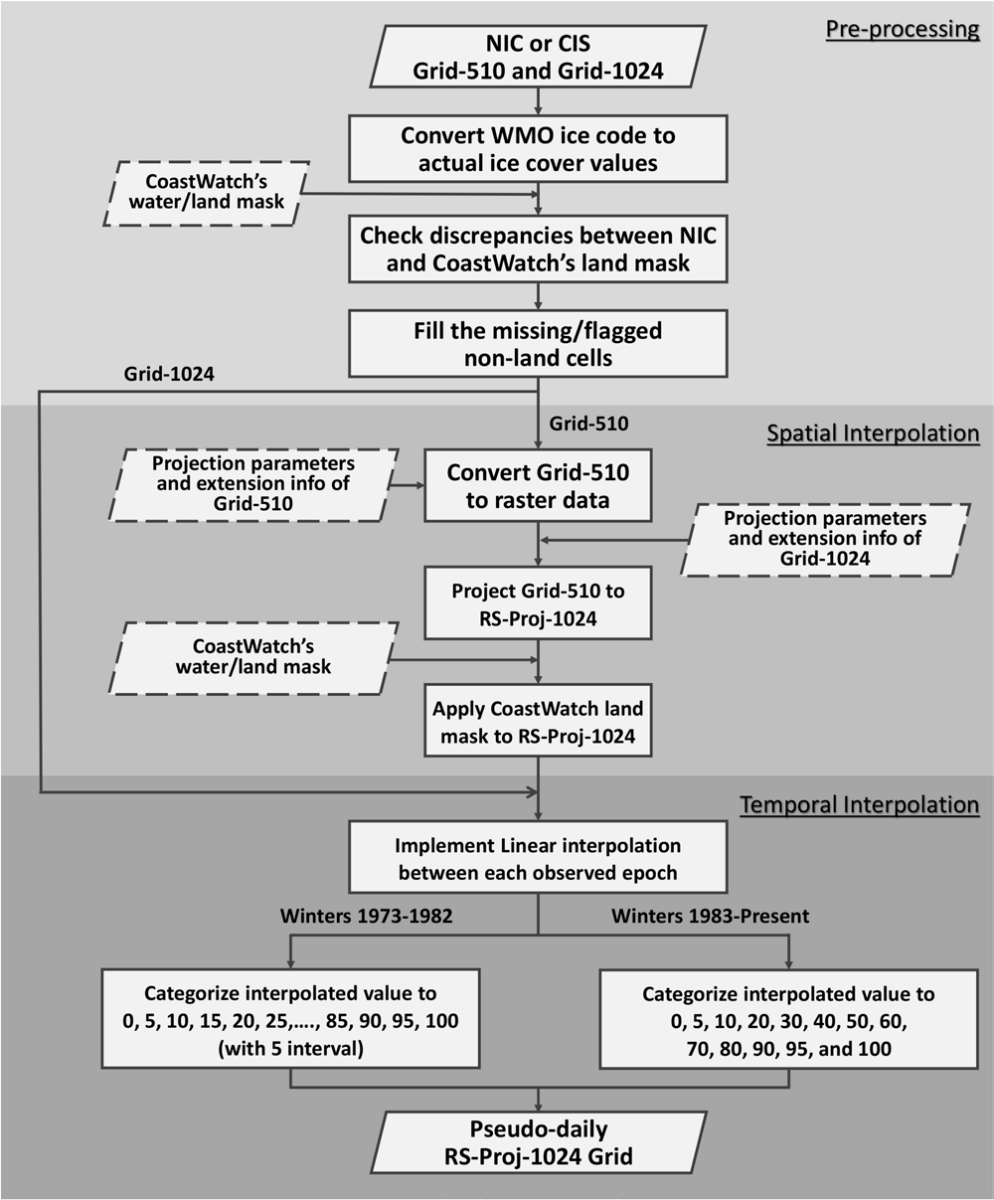
Flowchart of GLIC generation.

The Methods section is divided into three subsections: 1) Source Data and Pre-processing which briefly describes data format and pre-processing steps applied to ice cover data acquired from USNIC; 2) Spatial Interpolation for Winters 1973–2007 which describes how Grid-510 is re-projected and resampled to Grid-1024; 3) Temporal Interpolation for Winters 1973–2010 which describes the linear interpolation applied to Grid-1024 in the temporal domain for generating pseudo-daily GLIC. The flowchart for all procedures is shown Fig. 2.

### Source data & pre-processing

Ice cover data are projected on a 2D square grid, and values representing the percentage of ice cover for each grid cell are categorized in 21 levels (between 0 to 100 with intervals of 5) before winter 1983 and 13 levels (0, 5, 10, 20, 30, 40, 50, 60, 70, 80, 90, 95, and 100) since winter 1983. The cells representing land are assigned a value of −1. If grid cells are missing data, they are flagged with −99 (though we have been vigilant to prevent missing data from appearing in the final dataset to date.) To produce ice cover consistent with the data historically hosted by GLERL, the following steps are applied after downloading the ice chart from USNIC:

A. Convert the WMO^9^ ice codes to ice cover percentage values: In order to exchange the ice data with other countries, USNIC and CIS follow internationally recommended terminology and symbology established by WMO code to describe the ice cover condition. This non-intuitive representation of ice cover is converted to numeric form by describing ice cover as real percentages (0–100) of ice cover for each cell. The transformation from WMO codes to percentages is listed in Table 1.

**Table 1:** Transformation table between WMO ice code and ice cover percentages.

WMO Ice Code	GLERL Ice Cover Values (%)	WMO Ice Code	GLERL Ice Cover Values (%)
00	00	50	50
01	05	60	60
02	10	70	70
10	10	79	80
13	20	80	80
20	20	90	90
30	30	91	95
40	40	92	100
46	50	99	−99
B. Check for discrepancies between USNIC and CoastWatch water/land mask^10^: To be consistent with other GLERL CoastWatch gridded data products, NOAA Great Lakes CoastWatch water/land mask is applied to Grid-510 and Grid-1024. For cells that USNIC considers water but GLERL considers land, the value is simply set to land (−1). For cells that USNIC considers as land but GLERL considers water, the value is set to missing (−99) and handled in the next step.
C. Fill the missing non-land cells (−99): The missing values are estimated using the Nearest Neighbor Search (NNS) method, which searches for the nearest valid ice cover value and assigns it to the missing cell.

### Spatial interpolation for winters 1973–2007

In winter 2008, GLERL and USNIC upgraded the spatial resolution, and also changed the projection from Clarke_1866_Mercator to World_Mercator (Table 2). This change in projections updates the global ellipsoid from the Clarke 1866 to World Geodetic Survey 1984 (WGS84) increasing the locational accuracy of the grid. It should be noted that these are both global projections and are not the most accurate projection to be used at a regional scale, such as the Great Lakes basin. We have chosen to keep the ice data in the World Mercator projection to modify the original data as little as possible and maintain consistency among datasets (e.g., CoastWatch surface temperature). During the conversion year, winter 2007, USNIC provided both Grid-510 and a resampled version of Grid-1024. For consistency, we applied our spatial interpolation approach to winter 2007 Grid-510. However, we found that Grid-510 data of 2007/01/08 and 2007/01/22 is invalid, so instead we used temporal interpolation on these dates.

**Table 2:** Projection parameters for Grid-510 and Grid-1024.

	Grid-510	Grid-1024
Name	Clarke_1866_Mercator	World_Mercator
Projection	Mercator	Mercator
False_Easting	0	0
False_Northing	0	−24
Central_Meridian	−84.14	0
Standard_Parallel_1	45.04	0
Linear Unit	Meter (1.0)	Meter (1.0)
Geographic Coordinate System	GCS_Clarke_1866	GCS_WGS_1984
Angular Unit	Degree (0.0174532925199433)	Degree (0.0174532925199433)
Prime Meridian	Greenwich (0.0)	Greenwich (0.0)
**Datum**		
Spheroid	Clarke_1866	WGS_1984
Semimajor Axis	6378206.4	6378137
Semiminor Axis	6356583.8	6356752.314
Inverse Flattening	294.9786982	298.2572236
proj4 string	‘+ proj = merc + lon_0 = −84.14 + lat_ts = 45.04 + x_0 = 0 + y_0 = 0 + ellps = clrk66 + units = m + no_defs’	‘+ proj = merc + lon_0 = 0 + k = 1 + x_0 = 0 + y_0 = −24 + datum = WGS84 + units = m + no_defs'
**Extension**		
Left	−649446.2500	−10288021.9553
Right	666353.7500	−8444821.9553
Top	4606760.0000	6519174.1583
Bottom	3306260.0000	4675974.1583

**Fig. 3:**
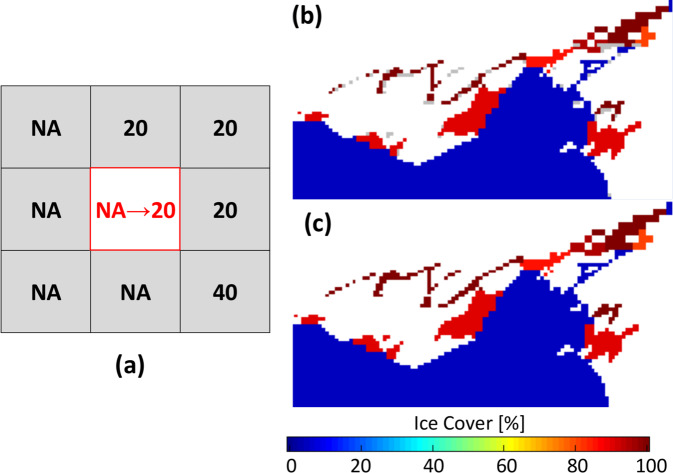
(**a**) Diagram for filling missing values, (**b**) Grey pixels are the locations of missing value cell, which are required to fill in the values, (**c**) The results after the filling step.

Through our comparison (see Technical Validation: Comparison of upscaling techniques), the RS-Proj method appears to preserve the accuracy and spatial features of Grid-510 data. Unlike the Kriging and bilinear interpolation, the RS-Proj method results have no artificial transition zones between high and low ice cover value. Compared with Wang *et al*.’s^7^ results, RS-Proj shows reduced shifting error (the error due to discrepancy of spatial projections between Grid-510 and Grid-1024). The detailed RS-Proj steps are:

A. Convert ASCII Grid-510 to raster: Assign projection system and extension information to Grid-510, to transform grid data to raster data. The description of projection for Grid-510 is given in Table 2.
B. Reproject and resample Grid-510 to Grid 1024: Create a new raster with Grid-1024 projection system (parameters listed in Table 2), and reproject Grid-510 to this raster using the “projectRaster” function in R. This function uses NNS method to resample the data from the source raster to the destination raster. The result of this step will be referred to as RS-Proj-1024.
C. Apply CoastWatch land/water mask to RS-Proj-1024: Due to slight differences between the land/water mask of Grid-1024 and Grid-510, the CoastWatch 1024 × 1024 land mask is applied to RS-Proj-1024. There are two scenarios in this step (both are handled similarly to the above land/water fill step in pre-processing):

- Over-water cell in RS-Proj-1024 being over-land cell in CoastWatch land mask: We re-assign “−1” (land area) to RS-Proj-1024.
- Over-land cell in RS-Proj-1024 being an over-water cell in CoastWatch land mask: We fill in the values, using is the most common value neighboring that pixel. For example, see Fig. 3a, the red box is a pixel which should have values in it, NA indicates no value for that pixel. In the search window (3 × 3 in this case), 20% is the most common value, so we assign 20% to the red box. If no valid values fall within the immediate neighbors, we enlarge the searching window iteratively until we find the valid ice cover values. An example of the filling step in are shown in Fig. 3b,c.

### Temporal interpolation for winters 1973–2010

Throughout the period of record, USNIC and CIS reported ice cover at varying frequencies (Fig. 1), which resulted in inconsistent data availability in time. However, daily data is desirable for many public and scientific applications (e.g. biological activity analysis, long-term weather monitoring, and route planning for commercial ships) as it allows querying and analysis of historical ice cover on any given day of year throughout the entire period of record. To fulfil these requirements, we produced pseudo-daily GLIC data. Each file that was generated via temporal interpolation is indicated as such in the header information. This allows users of the data to differentiate between observed and interpolated data. The temporal interpolation procedure is listed below:

A. Implement Linear interpolation between each observed grid: After spatial interpolation, RS-Proj-1024 grids (before winter 2008) and Grid-1024 (winter 2008 to 2010) are imported and used as a known data grid. The unknown ice cover between observed grids is estimated using linear interpolation method.
B. Convert interpolated value into a categorical value: To retain the original ice cover value representation, the interpolated value is then transformed to categorical percentage value. Before winter 1983, the values were categorized to 21 levels, and after winter 1983, the interpolated values were converted to 13 levels consistent with the levels described above in source data and pre-processing.

**Fig. 4:**
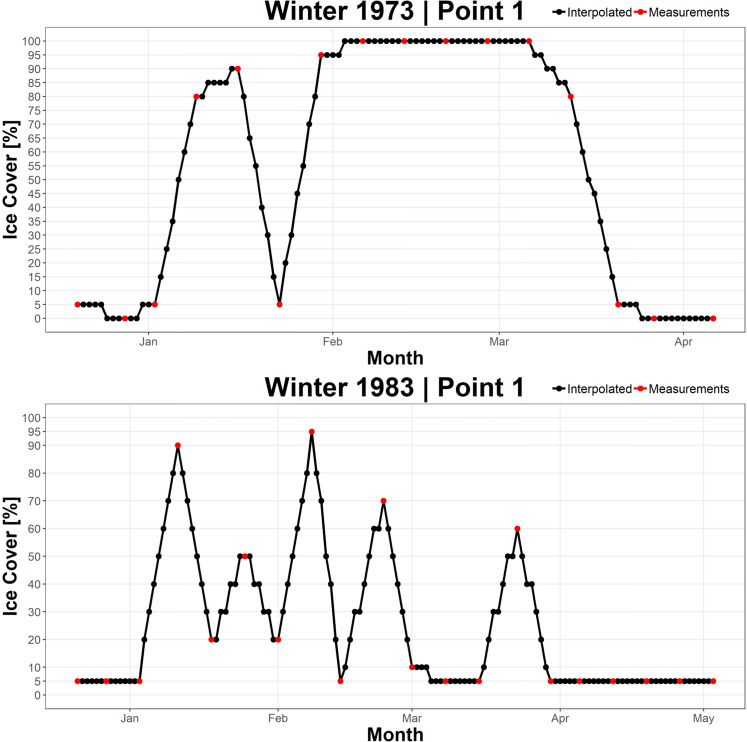
The time series of center points of validation region 1 (see Fig. 7) in winter 1973 and winter 1983. Red dots represent the original observations, black dots are temporal interpolated results.

To avoid large errors, extrapolation is not employed in this process (i.e. no data is generated outside of the observed time period). The temporal interpolation results (shown in Fig. 4) confirm that the ice cover values are assigned correctly to categorical levels after linear interpolation.

## Data Records

All GLIC data [Grid-510 (before winter 2008), Grid-1024 (since winter 2008), RS-Proj-1024 (before winter 2008), and pseudo-daily data (winter 1973 to 2010)] are available on the NOAA GLERL website^1^ and archived at the National Snow and Ice Data Center^11^ (NSIDC). These GLIC data are available as ASCII grid files for each day in the period of record. Ice charts are also available as jpgs for each day on which there was observation based data available from the USNIC or CIS.

## Technical Validation

### Comparison of upscaling techniques: kriging, bilinear, RS-SH and RS-Proj

Caution was taken in selecting the upscaling technique in order to preserve the accuracy and spatial features of the Grid-510 data. To ensure RS-Proj method is the best method, we present the skill of four spatial interpolation methods: (1) Kriging Interpolation with exponential function, (2) bi-linear spline, (3) a method we refer to as resampling-shifting (RS-SH) which involves duplicating each grid cell in both East-West and North-South directions and then shifting and subsetting the result (developed by Wang *et al*.^7^), and 4) our RS-Proj method.

**Fig. 5:**
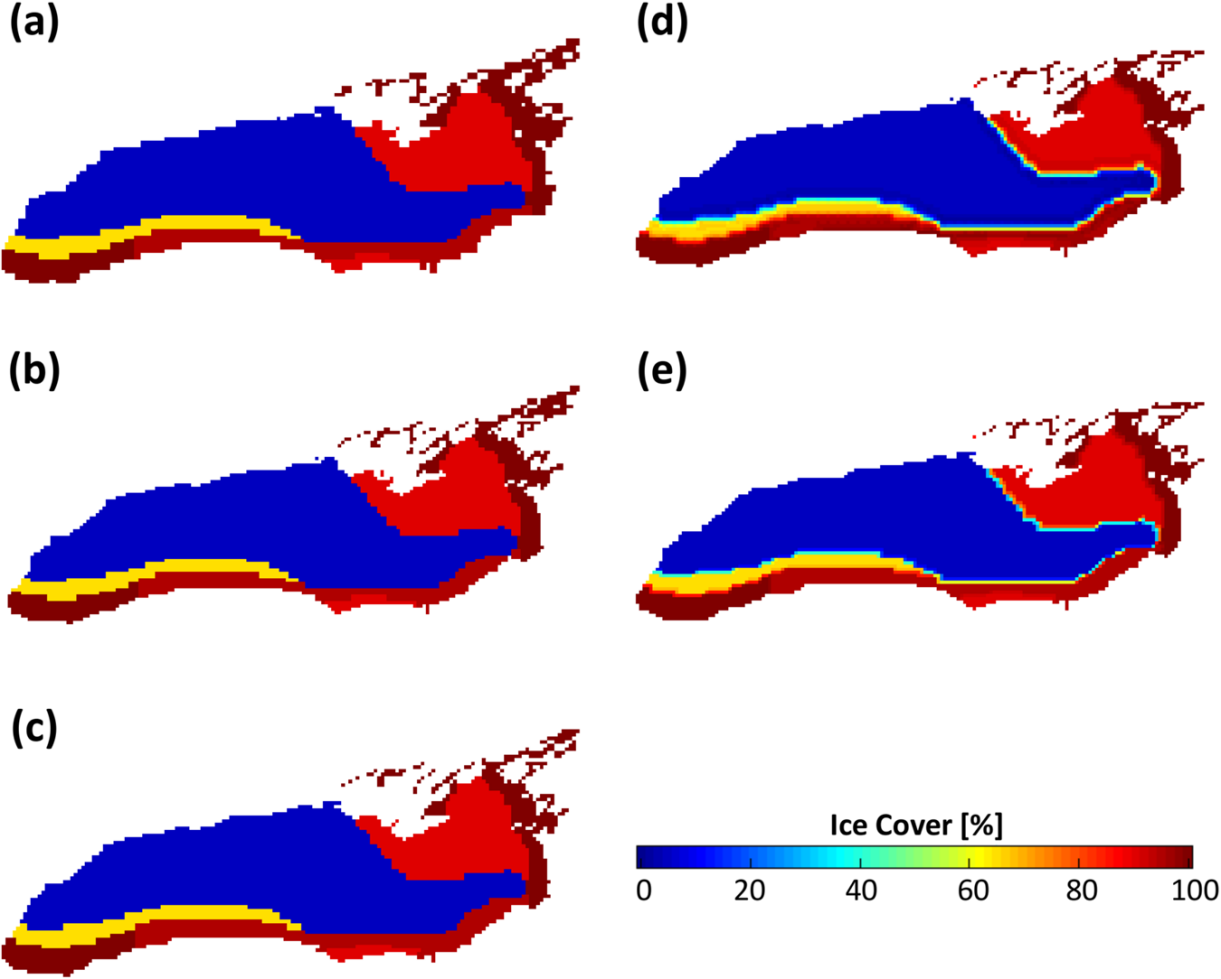
Comparison between various interpolation methods in Lake Ontario on 1978/03/08 (**a**) Grid-510 [original data], (**b**) RS-SH, (**c**) RS-Proj, (**d**) Kriging with exponential function (**e**) Bilinear spline.

Figure 5 demonstrates the comparison between the original Grid-510 data with the four upscaling techniques for Lake Ontario. Applying Kriging and bi-linear spline (Fig. 5d,e), results in artificial transition zones between high and low ice coverage areas, e.g., the green line between blue and red areas. By their nature, interpolation methods smooth data, which prevent sharp features from being well-represented in the output of the interpolation. This causes steep gradients in ice coverage to be reduced. To preserve spatial features accurately, Kriging and bilinear methods were deemed unacceptable for our process.

### Spatial comparison between RS-SH and RS-Proj

**Fig. 6:**
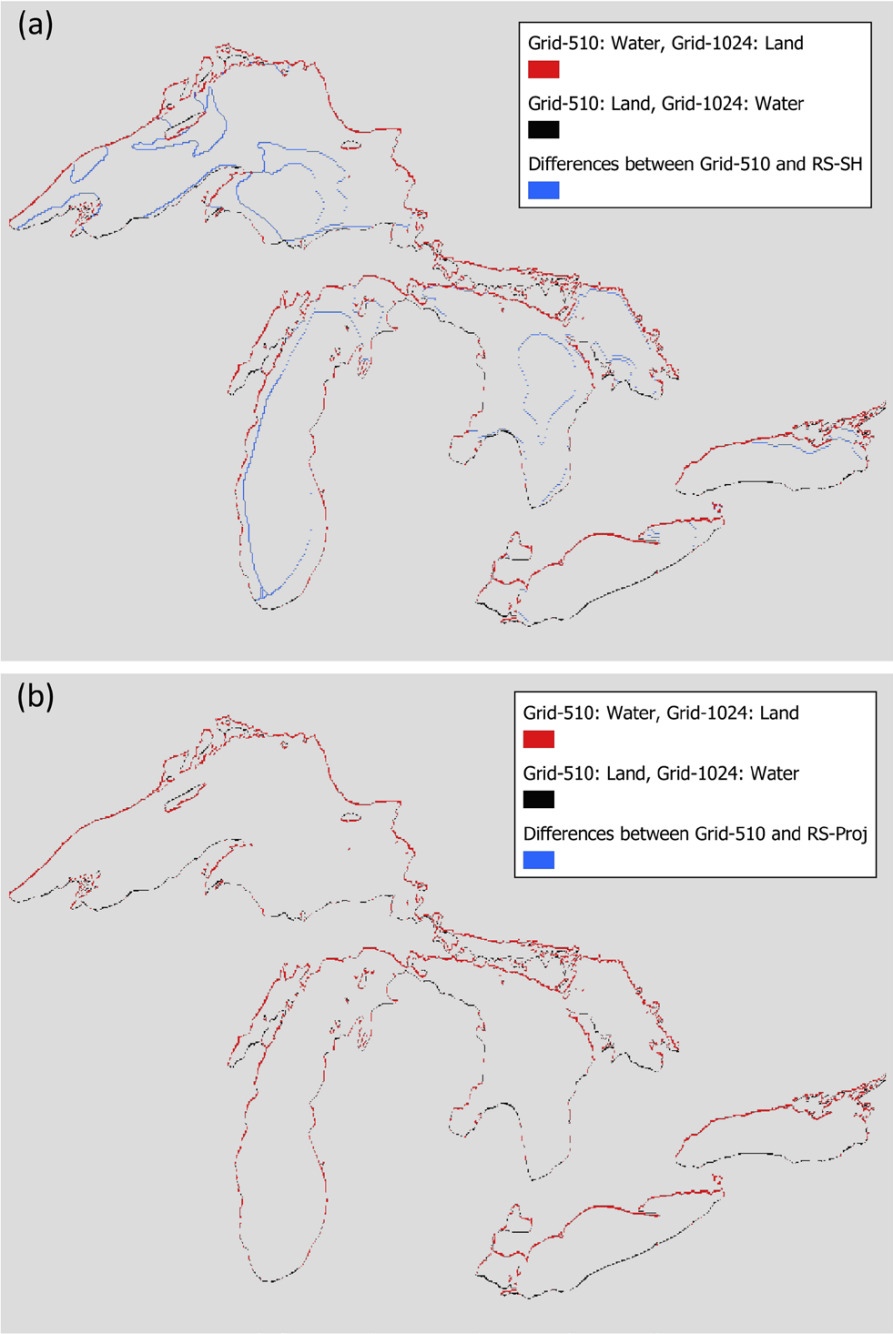
The comparison between original Grid-510, RS-SH and RS-Proj on 1981/02/04. (**a**) Difference between Grid-510 and RS-SH. (**b**) Difference between Grid-510 and RS-Proj. Red and black color grids represent the differences of land/water mask between Grid-510 and Grid-1024. Over-water grids in Grid-510 being over-land cells in Grid-1024 are represented by red color. In contrast, over-land cells in Grid-510 being over-water grids in Grid-1024 are filled by black color. Blue indicates the difference between Grid-510 and spatial interpolation results (RS-SH and RS-Proj) over-water grid.

Figure 5 shows that both RS-SH (Fig. 5b) and our selected RS-Proj (Fig. 5c) accurately maintain the spatial pattern. For further comparison and validation, we randomly select data from a single day (Feb 4th, 1981), and subtract RS-Proj and RS-SH from the original Grid-510 using the raster calculator function in QGIS. The results are shown in Fig. 6. It’s clear that RS-SH has a significant shifting error (blue cells in Fig. 6a) along sharp features while RS-Proj does not. This demonstrates that the RS-Proj method accurately transforms Grid-510 to Grid-1024. The error in the RS-SH is simply due to the design of this interpolation method. After duplicating each grid cell in both East-West and North-South directions, the new dimensions (1020 × 1032) do not match those of Grid-1024 and must be shifted and subset.

Both results contain spatial errors along the shoreline (red and black cells in Fig. 6) due to slight differences in water/land pixels for Grid-510 and Grid-1024 land mask^9^. This issue is unavoidable due to the discrepancies in shoreline definition at different scales. Both shorelines were derived directly from Advanced Very High Resolution Radiometer (AVHRR) satellite imagery.

### Time series comparison between Grid-510 and RS-Proj-1024

**Fig. 7:**
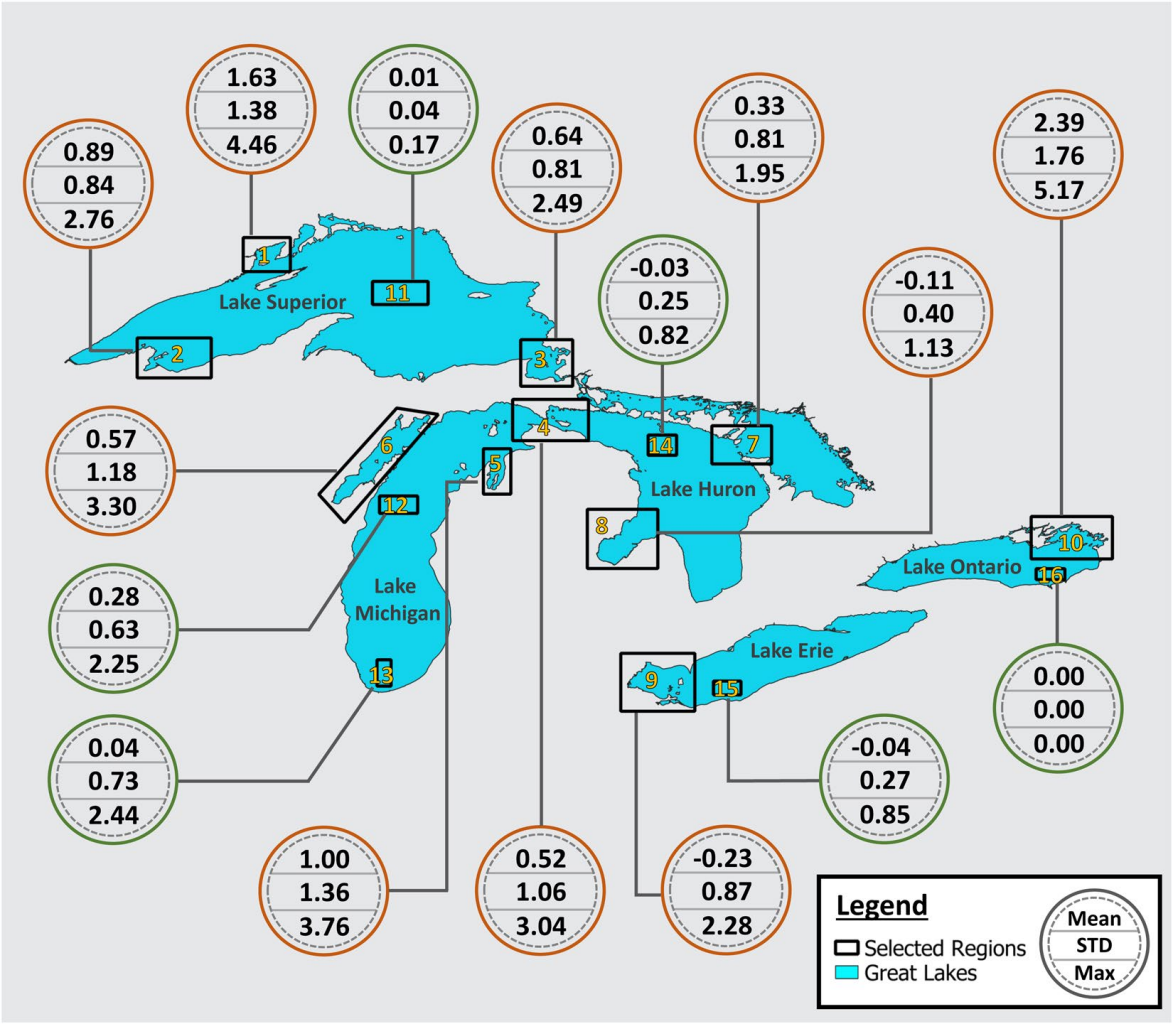
The location and extent of validation regions, and the statistics results of time series difference between Grid-510 and RS-Proj-1024 in each region. The orange and green circles represent the statistical results in Region 1–10 and Region 11–16, respectively. In each circle, the numbers stand for mean, STD and maximum (from top to bottom) of differences between time series of Grid-510 and RS-Proj-1024.

**Fig. 8:**
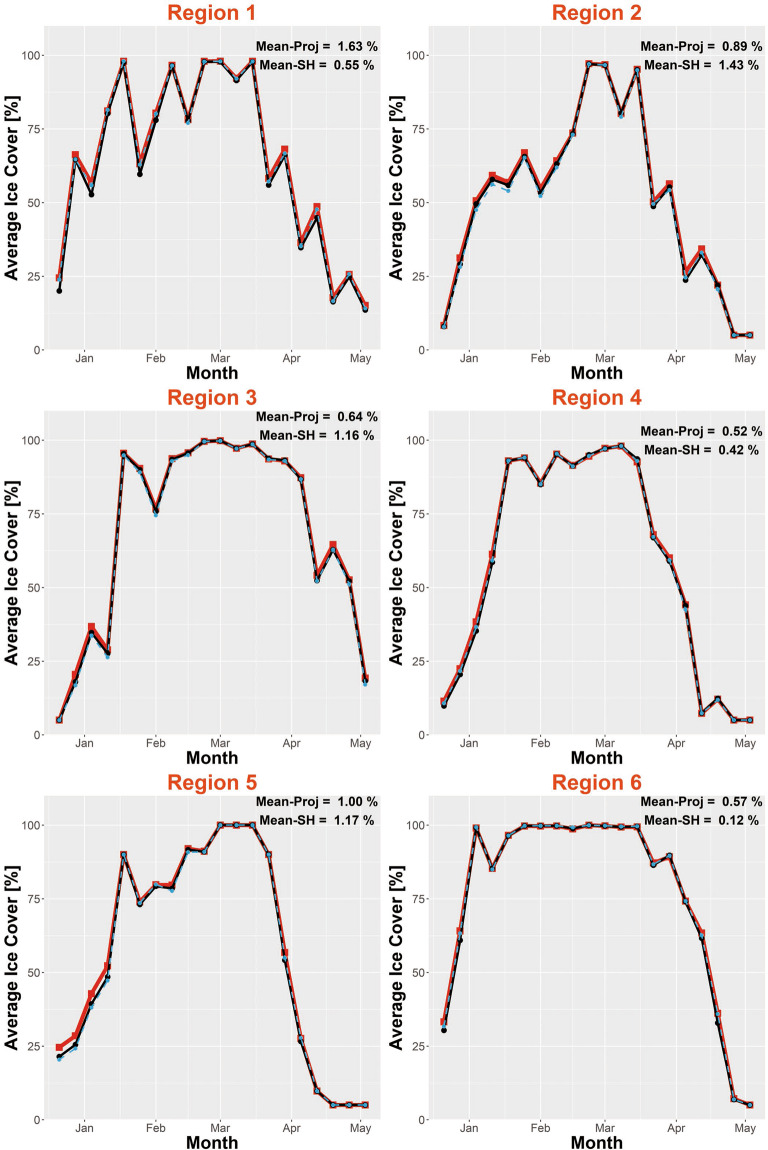
Time series of averaged ice coverage data in winter 1978 winter in Region 1~6. Regions position and area are shown in Fig. 7. Red line is the original Grid-510 file, black-dot line is the RS-Proj-1024 result, blue dashed line is the RS-SH result. *The Mean represents the mean of the difference between Grid-510 and RS-Proj-1024 (or RS-SH) in each region.

**Fig. 9:**
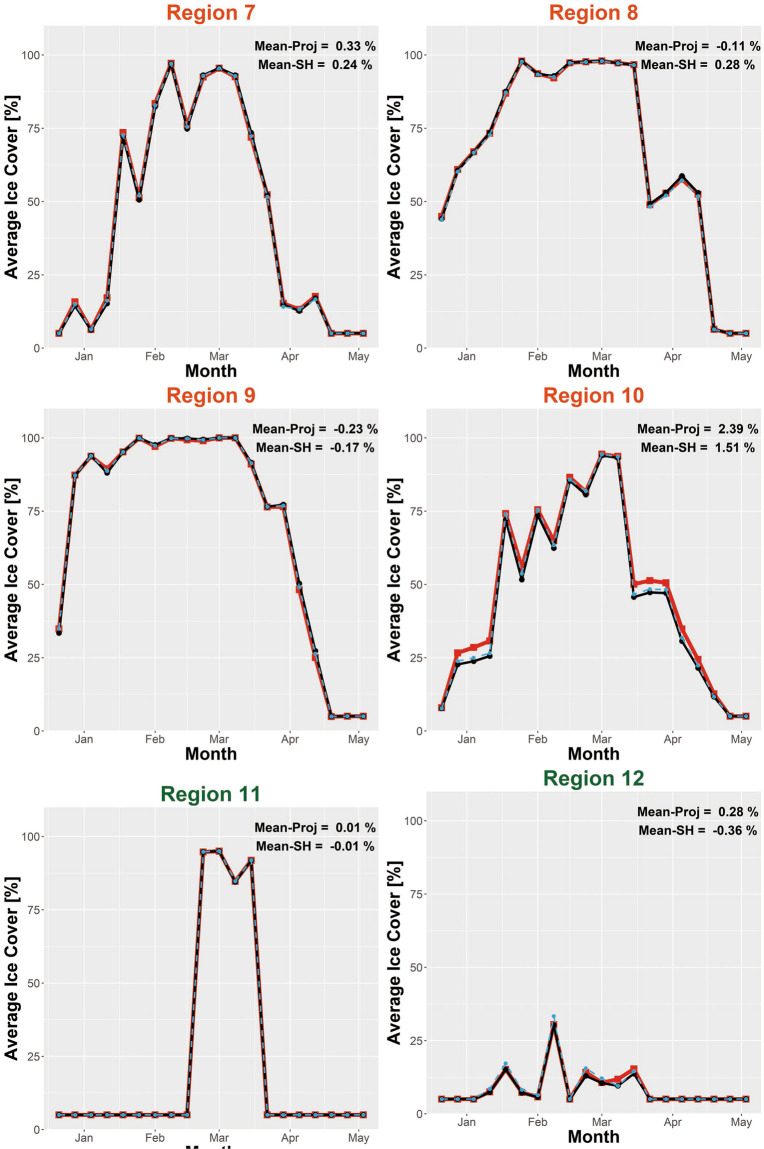
Time series of averaged ice coverage data in winter 1978 winter in Region 7~12. Regions position and area are shown in Fig. 7. Red line is the original Grid-510 file, black-dot line is the RS-Proj-1024 result, blue dashed line is the RS-SH result. *The Mean represents the mean of the difference between Grid-510 and RS-Proj-1024 (or RS-SH) in each region.

**Fig. 10:**
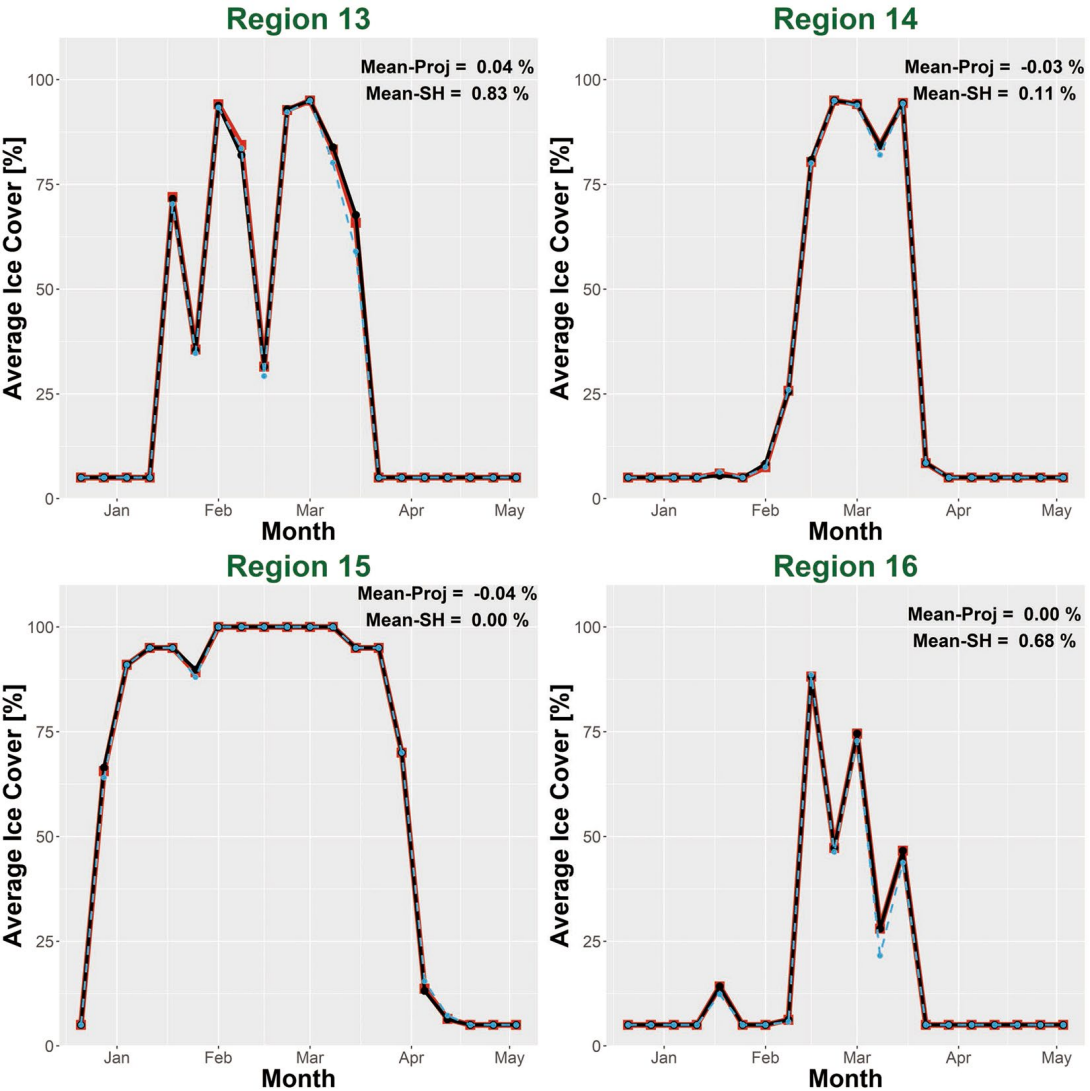
Time series of averaged ice coverage data in winter 1978 winter in Region 13~16. Regions position and area are shown in Fig. 7. Red line is the original Grid-510 file, black-dot line is the RS-Proj-1024 result, blue dashed line is the RS-SH result. *The Mean represents the mean of difference between Grid-510 and RS-Proj-1024 (or RS-SH) in each region.

Because the original and destination grids have different resolutions and dimensions, the raster calculation tool resamples Grid-510 (or RS-Proj-1024) to derive the spatial differences. Therefore, the previous validation method, spatial comparison, is not entirely reliable or perfect. Also, there is no overlapping Grid-510 and Grid-1024 data to validate our spatial interpolation result. As an additional validation method, we chose 16 subregions (shown in Fig. 7) to validate RS-Proj interpolation results. The subregions were intentionally selected to include both near-shore (Region 1–10) and open water (Region 11–16) areas. In each validation region, we averaged the respective ice coverage data for each sampling time, then compared the time series for RS-Proj-1024 and Grid-510 data. Figures 8–10 show the time series of RS-Proj-1024 (black line), RS-SH (blue dashed line) and Grid-510 (red line) in December 1977 to May 1978, which was a high ice cover year. The mean, standard deviation (STD), and max of differences between RS-Proj-1024 and Grid-510 in each region are listed in Fig. 7. From the time series plots, RS-Proj results compare favorably with the original Grid-510 data. 77.5% of the differences between Grid-510 and RS-Proj-1024 results are less than 1% ice concentration, and only 5.6% of the differences are larger than 3% ice concentration. The mean of all differences between Grid-510 and RS-Proj-1024 are less than 2.4%, with less than 1.8% STD. Compared with RS-SH, the means of difference between Grid-510 and RS-Proj-1024 are smaller in all regions except Region 15 (−0.04%). These results indicate the RS-Proj method is more reliable and accurate than RS-SH. The largest difference (5.2%) exists in Region 10, north shore of Lake Ontario. The discrepancy between Grid-510 and RS-Proj-1024 is mainly due to shoreline definition (see Fig. 6). As a result, the error increases through time and increases when there is a high percentage of ice near shore.

**Fig. 11:**
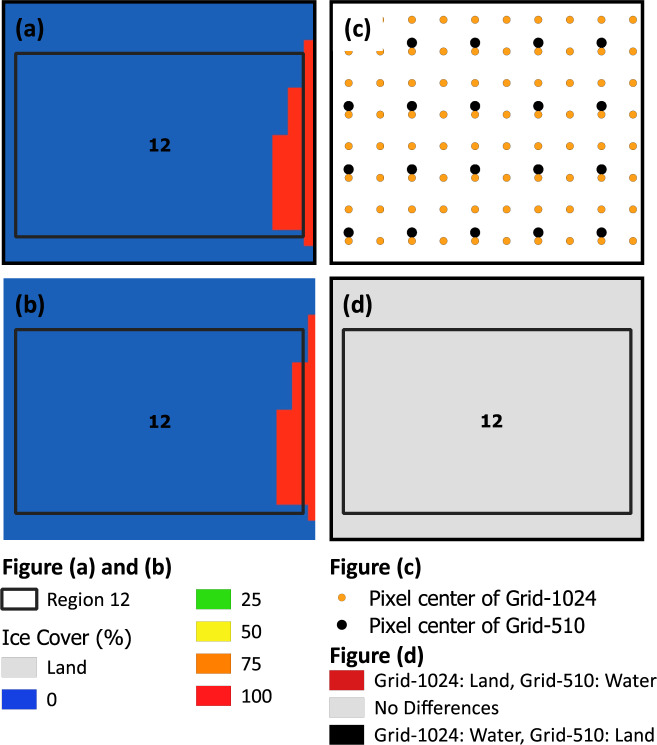
Comparison in Region 12 on 1978/03/08. (**a**) Grid-510, (**b**) RS-Proj-1024, (**c**) Pixel center differences between Grid-510 and RS-Proj-1024, (**d**) Differences between RS-Proj-1024 and Grid-510.

In offshore areas (Region 11–16), the spatial comparison of RS-Proj should be exactly the same as Grid-510, since there is no shoreline error in those areas. However, it should be noted that we did find some offshore RS-Proj-1024 data to be slightly different from Grid-510. For example, 2.3% difference in Region 12 can be seen on Mar 8^th^, 1978 (see Fig. 9). This is likely due to the fact that the grid center locations for Grid-510 and Rs-Proj-1024 are less than one pixel apart (Fig. 11c). This causes a slightly different area of pixels to be evaluated for Grid-510 vs RS-Proj-1024 (see Fig. 11a,b). Based on our comparison and validation, it is determined that RS-Proj is the best method for transforming Grid-510 to Grid-1024.

## Data Availability

We developed R scripts to compute the spatial and temporal interpolated ice cover values. Spatial interpolation for Grid-510 is processed by “Resampling_Raster.R”, and temporal interpolation for non-daily data is estimated by “Time_Interp.R”. Both scripts utilize RStudio version 1.1.463, and are available on the NOAA GLERL GitHub Repository at https://github.com/NOAA-GLERL/icegridresampling. This repository also contains sample scripts (Python, MATLAB and R) to demonstrate how to load the ASCII data into memory.

## References

[1] NOAA Great Lakes Environmental Research Laboratory. Historical Great Lakes Ice Cover. Data Archive https://www.glerl.noaa.gov/data/ice/#historical (2020)

[2] Simard, B. *et al*. *MANICE: Manual of Standard Procedures for Observing and Reporting Ice Conditions*. Catalogue No. En56-175 (Canadian Ice Service Meteorological Service of Canada, 2005).

[3] U.S. National Ice Center. Great Lakes Ice Analysis Product. https://www.natice.noaa.gov/products/great_lakes.html (2020)

[4] Norton, D. C. *et al*. *Great Lakes Ice Data Rescue Project*. Technical Memorandum No. 117 (NOAA Great Lakes Environmental Research Laboratory, 2000)

[5] Assel, R. A., Norton, D. C. & Cronk, K. C. *A Great Lakes Ice Cover Digital Data Set for Winters 1973*–*2000*. Technical Memorandum No. 121 (NOAA Great Lakes Environmental Research Laboratory, 2002)

[6] Assel, R. A. *Great Lakes Ice Cover Climatology Update: Winters 2003, 2004, and 2005*. Technical Memorandum No. 135 (NOAA Great Lakes Environmental Research Laboratory, 2005).

[7] Wang, J., Assel, R. A., Walterscheid, S., Clites, A. H. & Bai, X. *Great Lakes Ice Climatology Update, Winters 2006–2011, Description of the Digital Ice Cover Dataset*. Technical Memorandum No. 155 (NOAA Great Lakes Environmental Research Laboratory, 2012).

[8] Wang, J. *et al*. *Great Lakes Ice Climatology Update of Winters 2012–2017: Seasonal Cycle, Interannual Variability, Decadal Variability, and Trend for the period 1973–2017*. Technical Memorandum No. 170 (NOAA Great Lakes Environmental Research Laboratory, 2017).

[9] U.S. National Ice Center. World Meteorological Organization (WMO) Gridded Sigrid Format for Sea Ice. https://www.natice.noaa.gov/products/sigrid.html (2020)

[10] NOAA CoastWatch. Great Lakes CoastWatch Land Masks and Shoreline Data Files. https://coastwatch.glerl.noaa.gov/ftp/masks/ (2020)

[11] NOAA Great Lakes Environmental Research Laboratory (2020). NSIDC.

